# Evaluating alternate models to estimate genetic parameters of calving traits in United Kingdom Holstein-Friesian dairy cattle

**DOI:** 10.1186/1297-9686-44-23

**Published:** 2012-07-28

**Authors:** Sophie A E Eaglen, Mike P Coffey, John A Woolliams, Eileen Wall

**Affiliations:** 1 Animal & Veterinary Sciences Group, SAC, Roslin Institute Building, Easter Bush, Midlothian EH25 9RG, UK; 2 The Roslin Institute and R(D)SVS, University of Edinburgh, Easter Bush, Midlothian EH25 9RG, UK

## Abstract

**Background:**

The focus in dairy cattle breeding is gradually shifting from production to functional traits and genetic parameters of calving traits are estimated more frequently. However, across countries, various statistical models are used to estimate these parameters. This study evaluates different models for calving ease and stillbirth in United Kingdom Holstein-Friesian cattle.

**Methods:**

Data from first and later parity records were used. Genetic parameters for calving ease, stillbirth and gestation length were estimated using the restricted maximum likelihood method, considering different models i.e. sire (−maternal grandsire), animal, univariate and bivariate models. Gestation length was fitted as a correlated indicator trait and, for all three traits, genetic correlations between first and later parities were estimated. Potential bias in estimates was avoided by acknowledging a possible environmental direct-maternal covariance. The total heritable variance was estimated for each trait to discuss its theoretical importance and practical value. Prediction error variances and accuracies were calculated to compare the models.

**Results and discussion:**

On average, direct and maternal heritabilities for calving traits were low, except for direct gestation length. Calving ease in first parity had a significant and negative direct-maternal genetic correlation. Gestation length was maternally correlated to stillbirth in first parity and directly correlated to calving ease in later parities. Multi-trait models had a slightly greater predictive ability than univariate models, especially for the lowly heritable traits. The computation time needed for sire (−maternal grandsire) models was much smaller than for animal models with only small differences in accuracy. The sire (−maternal grandsire) model was robust when additional genetic components were estimated, while the equivalent animal model had difficulties reaching convergence.

**Conclusions:**

For the evaluation of calving traits, multi-trait models show a slight advantage over univariate models. Extended sire models (−maternal grandsire) are more practical and robust than animal models. Estimated genetic parameters for calving traits of UK Holstein cattle are consistent with literature. Calculating an aggregate estimated breeding value including direct and maternal values should encourage breeders to consider both direct and maternal effects in selection decisions.

## Background

Calving is a key event on a dairy cattle farm and successful calvings are important to financial success of the farm. Calving complications lead to an increase in veterinary and labour costs and a decrease in revenue (loss of animals and/or reduced subsequent performance) [1-3]. Furthermore, difficult calvings compromise animal welfare and thereby consumer acceptability of dairy production systems [4].

In recent years, dairy cattle breeders have shown an increasing interest in selection for functional traits [5] and gradually the focus of selection is shifting from traits that increase profit towards traits that reduce costs [6]. For example, easy parturition and calf viability are economically important traits but are not classical production traits. Since genetic selection could improve calving performance, it is important to include calving traits in genetic evaluations, although their implementation is not straightforward.

Calving ease (CE) and stillbirth (SB) are phenotypes that are generally scored on categorical or binary scales which make them sensitive to subjectivity, especially CE [1]. Furthermore, heritabilities of these traits are generally low [7] and thus much data is needed to obtain sufficiently accurate estimates that have an impact on selection indices. But above all, calving trait phenotypes are affected by two separate components, the calf’s contribution (direct effect; e.g. arising from size, hormonal balance, weight) and the dam’s contribution (maternal effect; e.g. arising from pelvic measurements, ability to respond to parturition signalling), and this complicates statistical analysis [8-10]. In quantitative genetics, the objective is to separate additive genetic variances and co-variances from other sources of variance. The statistical model fitted for calving traits should therefore allow the separation and estimation of both direct and maternal effects but there is no consensus on which is the most accurate model to achieve this objective. Various statistical models have been reported throughout the years, each aimed at improving one aspect of their predictive ability. Consequently, many different statistical models are used in routine genetic evaluations to estimate genetic parameters for calving traits [11], while for production traits there is a greater consensus across countries [5]. For CE, statistical models range from sire-maternal grandsire (S-MGS) models to animal models in univariate or multi-trait form that either allow a direct-maternal genetic covariance or fix this covariance to zero [11].

Overall, statistical models can account for direct and maternal effects in two ways i.e. animal models that fit calf and dam effects directly and S-MGS models that fit direct and maternal effects through the sire of the calf and dam, respectively [11]. Each of these then has a family of implementation depending on how traits are defined and modelled e.g. univariately or multi-trait, which leads to the divergence in models seen today. The genetic covariance between direct and maternal effects plays a key role in the interpretation of estimated genetic parameters and the prediction of response to selection. For example, in the case of CE, this parameter represents the genetic relationship between the animals’ genetic predisposition for ease of being born and ease of giving birth.

This genetic covariance is however suggested to be sensitive to estimation bias [12-14]. Thus, estimates of direct-maternal genetic correlations reported for calving traits, which are often negative and of moderate to high magnitude, are questioned [15]. Theories on the source of bias in estimates of direct-maternal genetic correlations range from ignored environmental covariances [14,16,17] to unaccounted contemporary groups [15,18,19]. Estimation of the direct-maternal covariance and remaining genetic components is said to be further improved by using a multi-trait model instead of a univariate model [20,21]. Since CE and SB are highly correlated and show low heritabilities, it has been suggested that a multi-trait model that incorporates a highly heritable and correlated indicator trait such as birth weight, calf size and/or gestation length (GL), would lead to a more optimal analysis [20-22]. In addition to models incorporating indicator traits, it has also been proposed that the extension of univariate models to a multi-trait model between parities would be useful because of the potential genetic distinctiveness of calving traits in first and later parities. This implies that models fitting first and later parities as correlated traits are theoretically more correct than models that fit parity as a non-genetic effect [23-25].

However, given the high standard errors of the estimated maternal variances and direct-maternal covariances, it appears that statistical models used to analyse calving traits can still benefit from optimization. Previous studies on the estimation of genetic parameters for calving traits have been comparing models. However, in most cases, studies limit themselves to single comparisons, such as univariate versus multi trait models within either a sire or animal model framework [21,24]. The objective of this study was to estimate the genetic parameters of calving traits for United Kingdom (UK) Holstein-Friesian cattle with a range of present-day statistical models for national genetic evaluations of calving traits. A comparison was made between S-MGS versus animal models and univariate versus multi-trait models between traits and parities.

GL was added to the multi-trait model to study any benefits of this indicator trait to the predictive ability of the model. Specific attention was given to the potential bias of the sensitive genetic correlation between direct and maternal effects and, lastly, a ‘total heritable variance’ for calving traits was estimated, combining direct and maternal variance components. This parameter was recently reported for the analysis of traits affected by indirect genetic effects [26] and is suggested to represent the total amount of total genetic variance available for response to selection. Its practical use for calving traits is discussed.

## Methods

### Data description

Data on calving in Holstein Friesian cattle was provided by two milk recording organisations (MRO) in the UK i.e. the Cattle Information Service (CIS) and National Milk Records (NMR). GL was calculated from insemination and calving dates and restricted to 265–295 days. Only records on single born calves were used. Datasets from first and later parity records contained 43 135 and 330 753 records respectively, spanning the years 1995 to 2009. This data was checked for inconsistencies in parity, breed, calving date and age at calving. Incorrect records were discarded, as were contemporary groups with fewer than two records (herd, herd-year, sire and maternal grandsire). Age at calving was restricted to 18–48 months for heifers and 30–70 months for cows. To avoid herds where farmers unrealistically recorded the same CE score for most or all contemporary groups of calvings, the standard deviation of CE score within herd-year was calculated. Herd-year classes with standard deviations of zero were deleted when this was considered statistically improbable (97.5% confidence interval) given the herd size and CE score distribution. Sex of stillborn calves was not recorded by one of the data sources. As sex has been shown to have a considerable effect on SB [7] data from this specific data source was deleted for the univariate and bivariate between-parity analyses on SB. For all univariate analyses on CE and GL, the data from this source for a stillborn calf was set as missing but the remaining data obtained by the specific data source was used. The final dataset consisted of 30 640 first parity records originating from 2 098 herds representing 2 012 (service) sires and 4 783 maternal grandsires (MGS). The accompanying pedigree consisted of ~ 200 000 individuals and was 5 generations deep. Since the later parity dataset created computational problems when fitting the animal model, it was reduced by only retaining records that were of 2^nd^ and 3^rd^ parity and had values for each of the three traits (prior to edits on SB data described earlier). Furthermore, the thresholds for the minimum size of herd-year and sire contemporary groups were increased to 7 and 5 records, respectively. Finally, the final later parity dataset consisted of 54 744 records, originating from 2 108 herds, 1 918 sires and 5 886 maternal grandsires, with an accompanying pedigree of ~ 290 000 individuals and 4 generations deep.

CE was scored on a categorical scale, which ascends in calving difficulty and differs between data sources. Scores were therefore harmonised and transformed into values on the underlying normal distribution (average liability value) within data source and parity prior to analysis. Detailed information on the recording system and transformation of CE scores is reported in Eaglen et al. [3]. SB was recorded as a binary trait with a 0 value if alive and 1 if stillborn. Frequency distributions per data source within edited datasets, GL means, SB frequencies and other descriptive statistics of the data are given in Table 1.

**Table 1:** Descriptive statistics of the data

**Variable**	**Dataset**		
	**First parity**	**Later parities**	**Across parities**
Number of records	30 640	54 744	83 053
Number of dams	30 640	51 658	79 967
Number of dams with own birth record^1^	2 411	10 899	13 310
Number of sires	2 012	1 918	2 827
Number of maternal grandsires	4 783	5 886	8 291
Female calves	67.12%	46.08%	54.6%
Male calves	32.88%	53.92%	46.4%
CE, frequency^2,3^	71.67%	83.12%	79.03%
	24.33%	14.99%	18.54%
	3.33%	1.51%	2.20%
	0.67%	0.38%	0.50%
GL (days), mean ± s.e.^3^	280.69 ±4.97	281.35 ± 4.89	281.17 ± 4.93
SB, frequency^3^	11.6%	4.3%	6.0%

^1^The total number of records reported excludes this subset of records; ^2^1 = easy (non-assisted), 2 = moderate assistance (veterinarian called as precaution), 3 = difficult, 4 = very difficult with veterinary assistance; ^3^CE = calving ease, GL = gestation length, SB = stillbirth, s.e. = standard error.

### Statistical analyses

#### Direct and maternal genetic effects

In this study, models will follow Willham’s model [8] where the phenotype observed on an individual calf *i* for CE, SB and GL is modelled by

(1) $$P_{i}=A_{d,i}+E_{d,i}+A_{m,j}+E_{m,j}\text{.}$$

Thus, during the life of *i*, the direct additive effect (A_d,i_) is expressed at the start of life while, if *i* is a female, the maternal additive effect of *i* (A_m,i_) is expressed whenever she calves. E_D,i_ and E_M,j_ are the direct environmental effect, property of calf *I*, and maternal environmental effect, property of dam *j*, respectively.

#### Statistical models

In all cases, optional fixed effects and potential interaction effects were tested for significance in SAS V9.1 (P < 0.05) [27] and then the variance components were calculated using REML, with ASREML version 3.0 [28]. Sex by parity and sex by age interaction effects were not significant. Prior to using multi-trait models involving GL, the relationship of GL with CE and SB was examined according to Hansen et al. [20] to ensure that the use of traditional bivariate models was appropriate given their assumption of linearity.

Furthermore, the limitations of analysing categorical traits, such as CE and SB, with linear models are well known. Multiple model assumptions are violated due to the fact that values of categorical data are bounded within certain limits e.g. 0 to 1 or 1 to 4. Therefore, generalized linear mixed models (GLMM) such as threshold models can be more appropriate for the analysis of categorical traits since scores are transformed by the model into values on an underlying continuous liability scale. This study aimed to evaluate statistical models with the ultimate goal of implementation in national genetic evaluations of CE, SB and GL. Although threshold models are implemented in routine national genetic evaluations in France and the USA [29,30], in the UK and most other countries, calving traits are evaluated with a linear model. Therefore, we chose to evaluate several modelling possibilities within linear rather than threshold models. In the discussion section of this paper, we will elaborate further on this choice.

#### Univariate animal and S-MGS models

To study the difference between animal and S-MGS models, first parity data were analysed by linear univariate models. Direct and maternal genetic effects were incorporated by fitting genetic effects for calf and dam for the animal model and sire and maternal grandsire for the S-MGS model:

(2) $$y=Xb+Z_{\text{d}}a_{\text{d}}+Z_{\text{m}}a_{\text{m}}+Z_{\text{hy}}h_{\text{hy}}+e\text{.}$$

In Equation 2, **y** is a vector representing the observations for CE, SB or GL; **X**, **Z**_d,_**Z**_m_ and **Z**_hy_ are known incidence matrices for non-genetic, and direct and maternal genetic and herd-year effects, respectively; **b** is a vector of non-genetic effects, **a**_d_ is a vector of the random direct additive-genetic effects of the calf (sire), **a**_m_ is a vector of the random maternal additive- genetic effects of the dam (maternal grandsire), **h**_hy_ is a vector of random herd-year effects and **e** is a vector of residuals. Vectors **a**_d_ and **a**_m_ were assumed to follow a multivariate normal distribution, with MVN(0, **G** = **G**_**0**_ ⊗ **A)** where, **G**_**0**_ was a 2 × 2 direct-maternal (S-MGS) variance-covariance matrix,⊗ is the Kronecker product of matrices, and **A** was the relationship matrix. **e** was assumed to be MVN(0, **I** σ_e_^2^), where **I** denotes the identity matrix and σ_e_^2^ the residual variance. Non-genetic effects in the models included sex of the calf, herd, sire breed (only for GL), year and month of calving, the interaction of year and month of calving; age of the dam (months) treated as a covariate and the interaction of herd and year of calving treated as a random factor. S-MGS models yield sire and maternal grandsire (co)variances (*σ*_*sire*_^2^,*σ*_*mgs*_^2^,*σ*_*sire*,*mgs*_) which were subsequently transformed algebraically into direct and maternal (co)variances (*σ*_*Ad*_^2^,*σ*_*Am*_^2^,*σ*_*Adm*_) according to

(3) $$\begin{array}{l} \sigma_{\mathrm{Ad}}^{2}=4\sigma_{\mathrm{sire}}^{2}\\ \sigma_{\mathrm{Adm}}=4\sigma_{sire,mgs}-2\sigma_{\mathrm{sire}}^{2}\\ \sigma_{\mathrm{Am}}^{2}=4\sigma_{\mathrm{mgs}}^{2}+\sigma_{\mathrm{sire}}^{2}-4\sigma_{sire,mgs}\text{.} \end{array}$$

#### Bivariate models between traits

Bivariate animal models were fitted pairwise among CE, SB and GL, separately for first and later parity data:

(4) $$\begin{array}{l} \left[\begin{array}{c} y_{1}\\ y_{2} \end{array}\right]=\left[\begin{array}{cc} X_{1} & 0\\ 0 & X_{2} \end{array}\right]\left[\begin{array}{c} b_{1}\\ b_{2} \end{array}\right]+\left[\begin{array}{cc} Z_{d\_1} & 0\\ 0 & Z_{d\_2} \end{array}\right]\left[\begin{array}{c} a_{d\_1}\\ a_{d\_2} \end{array}\right]\\ +\left[\begin{array}{cc} Z_{m\_1} & 0\\ 0 & Z_{m\_2} \end{array}\right]\left[\begin{array}{c} a_{m\_1}\\ a_{m\_2} \end{array}\right]+\left[\begin{array}{cc} Z_{\mathrm{hy}} & 0\\ 0 & Z_{\mathrm{hy}} \end{array}\right]\left[\begin{array}{c} h_{hy\_1}\\ h_{hy\_2} \end{array}\right]+\left[\begin{array}{c} e_{1}\\ e_{2} \end{array}\right]\text{.} \end{array}$$

In this model, vectors and incidence matrices correspond to those in the univariate animal model (Equation 2) and subscripts 1 and 2 denote traits. Non-genetic effects for later parities were the same as for univariate first-parity models, with the addition of a interaction between age of dam and parity treated as a fixed factor, and a random permanent environmental effect (**Z**_pe_**pe**_pe_). The covariance matrix of the genetic terms equalled, **G** = **G**_**0**_ ⊗ **A** where **G**_**0**_ was a 4 × 4 symmetrical direct-maternal variance-covariance matrix

(5) $$\text{Var}\left[\begin{array}{c} a_{d\_1}\\ a_{d\_2}\\ a_{m\_1}\\ a_{m\_2} \end{array}\right]=\left[\begin{array}{cccc} \sigma_{Ad\_1}^{2} & & & \\ \sigma_{{Ad}_{1,2}} & \sigma_{Ad\_2}^{2} & & \\ \sigma_{Ad\_1,m\_1} & \sigma_{Ad\_2,m\_1} & \sigma_{Am\_1}^{2} & \\ \sigma_{Ad\_1,m\_2} & \sigma_{Ad\_2,m\_2} & \sigma_{{Am}_{1,2}} & \sigma_{Am\_2}^{2} \end{array}\right]A.⊗$$

Residuals, **e**, and permanent environmental effects, **pe**_pe_, were assumed to be MVN(0, **R**_e_*σ*_*e*_^2^), and MVN(0, **R**_pe_*σ*_*pe*_^2^), where **R**_e_ and **R**_pe_ denote the residual and permanent environmental 2 × 2 variance covariance matrices and *σ*_*e*_^2^and *σ*_*pe*_^2^ were the residual variance and permanent environmental variance.

#### Bivariate models between parities

To study the genetic correlation between calving traits in first and later parities, bivariate S-MGS models were fitted with first and later parities (2^nd^ and 3^rd^ parities combined) treated as correlated traits. The model equalled equation 4, with **y**_i_ a vector representing the observations for each trait in first (y_1_) and later parities (y_2_). Random genetic effects were fitted for the sire and maternal grandsire. The fixed and random non-genetic effects were the same as in the univariate animal model. Estimates of sire and maternal grandsire variances were transformed into direct and maternal effects according to equation 3.

Direct and maternal heritabilities (*h*_*d*_^2^ and *h*_*m*_^2^) were estimated by:

(6) $$h_{d}^{2}=\sigma_{\mathrm{Ad}}^{2}/(\sigma_{\mathrm{Ad}}^{2}+\sigma_{\mathrm{Adm}}+\sigma_{\mathrm{Am}}^{2}+\sigma_{e}^{2})$$

and

(7) $$h_{m}^{2}=\sigma_{\mathrm{Am}}^{2}/(\sigma_{\mathrm{Ad}}^{2}+\sigma_{\mathrm{Adm}}+\sigma_{\mathrm{Am}}^{2}+\sigma_{e}^{2})\text{,}$$

where *σ*_*Ad*_^2^ and *σ*_*Am*_^2^ are the direct and maternal additive genetic variances, *σ*_*Adm*_ is the additive direct maternal covariance and *σ*_*e*_^2^ is the environmental variance. To allow easy comparison with other studies, herd-year variances and permanent environmental variances were not included in the phenotypic variance but are provided in Additional file 1: Tables S1 and S2. The heritabilities and genetic direct-maternal correlations were estimated more than once by the several bivariate models and these were pooled in meta- analyses according to Corbin et al. [31]

#### Direct-maternal genetic covariance

A negative direct-maternal relationship would be worrying for the dairy cattle industry since it suggests that selecting a sire that is genetically superior for ease of birth may later cause a problem when its daughters calve. Koch [14] showed that, when ignored or assumed to be zero, a direct-maternal environmental covariance (cov(E_d,i,_ E_m,i_)) can cause bias in the estimated genetic parameters. Although it is possible to fit a correlation structure in the residual to avoid this problem, computational complexity is then substantially increased. Therefore, in this study, we chose to avoid this potential bias by removing from the data all individuals that appeared as both calf and dam. Residuals of these specific records would otherwise be correlated [16]. Then to evaluate the bias, animals were reintroduced and the analyses were repeated. Throughout the paper, animal model 1 (A1) represents the animal model which was used to analyse the reduced data, whereas animal model 2 (A2) represents the animal model used to analyse the total data.

#### Total heritable variance

Additive genetic variances are estimated to evaluate the genetic differences between animals that can be used to generate a response to a chosen selection strategy. Equation 8 demonstrates that in the case of maternally affected traits, there are two additive genetic variances that can respond to selection. Analogous to the additive direct genetic variance, the additive maternal genetic variance is equivalent to the variance of maternal breeding values of individuals in the population, under random mating. The presence of two genetic variances responding to selection raises the question of a ‘total’ additive variance. According to Bijma et al. [26], the total breeding value of an individual for a maternally affected trait can be expressed as the sum of its direct breeding value (*A*_*d*,*i*_) and its maternal breeding value (*A*_*m*,*i*_), which is referred to as the *TBVi*

(8) $$TBV_{i}=A_{d,i}+A_{m,i}$$

from which the total heritable variance follows as:

(9) $$\sigma_{\text{TBV}}^{2}=\sigma_{\mathrm{Ad}}^{2}+2\sigma_{\text{Adm}}+\sigma_{\mathrm{Am}}^{2}\text{.}$$

In this context, the *σ*_*TBV*_^2^ represents the total genetic variance available for response to selection, with response predicted by *R* = *ιρ*_*M*_*σ*_*TBV*_^2^ where *ι* is the selection intensity and *ρ*_*M*_ is the accuracy of selection [16]. This is distinct from the total heritable variance reported by Willham [8], Meyer [13] and Koch [14], which refers to mass selection, as explained in Eaglen et al. [16]. Given the current selection strategies based on PTA in dairy cattle, we estimated and explored *σ*_*TBV*_^2^ as described by Bijma et al. [26].

## Results and discussion

Table 1 presents the descriptive statistics of the data. It shows that in the UK, approximately 20% of the calvings required assistance of some sort. Incidence of calving assistance was higher in first than in later-parity calvings, which agrees with the general consensus that calving complications are of more concern in heifers than in cows [7]. Moreover, severe calving difficulty was experienced by approximately 4% and 2% of heifers and cows respectively. These are in line with international prevalences of calving difficulty in the Holstein breed [4] although comparison is not straightforward since the scoring system of CE allows for a large variety of score definitions [7]. The incidence of SB in first and later parities (Table 1) agrees with incidences reported by Hansen et al. [20] and Jamrozik et al. [32]. Table 1 also shows that there were fewer males than females in the first-parity dataset, which could indicate a bias in data recording due to the difference in value between a bull and a heifer calf in dairy cattle. Since the calving of bull calves is known to be more difficult [16], it is possible that CE is under-reported. However, all studies using field records for CE data in dairy cattle will likely suffer from the same problem. The frequencies of female and male calves were more equal in later parities.

Given the amount of results obtained in this study, it was decided to separate the biological findings (genetic parameters), in Table 2, 3, 4 and 5, from the findings on the model comparisons. To aid in the comparison of different models, accuracies of predicted transmitting abilities (PTA) for 25 randomly selected young and older sires were calculated by their prediction error variances (PEV); PEV = (1-r^2^)*σ*_*Ad*_^2^ (Table 6). The PEV are provided in Additional file 1: Table S3. Throughout the study, the default model fitted was A1. When results of other models are discussed, this is indicated.

**Table 2:** **Genetic parameters**^**1 **^**for calving ease, stillbirth and gestation length from first parity bivariate animal models**

**Trait**^**2**^	***h***^**2**^	**Trait**^**2**^				
		**DSB**	**DGL**	**MCE**	**MSB**	**MGL**
DCE	0.12 (0.02)*	0.84 (0.18)*	0.18 (0.10)	−0.53 (0.13)*	0.28 (0.23)	0.02 (0.19)
DSB	0.02 (0.01)		−0.06 (0.27)	0.97 (0.23)*	0.37 (0.56)	−0.15 (0.14)
DGL	0.57 (0.05)*			0.09 (0.14)	−0.30 (0.23)	−0.23 (0.11)
MCE	0.05 (0.01)*				0.85 (0.13)*	−0.15 (0.12)
MSB	0.03 (0.01)*					0.65 (0.32)*
MGL	0.07 (0.02)*					

*P < 0.05; ^1^heritabilities and genetic correlations ^2^DCE = direct calving ease, DSB = direct stillbirth, DGL = direct gestation length, MCE = maternal calving ease, MSB = maternal stillbirth, MGL = maternal gestation length; standard errors are indicated in brackets.

**Table 3:** **Genetic parameters**^**1 **^**for calving ease, stillbirth and gestation length from later parities bivariate animal models**

**Trait**^**2**^	***h***^**2**^	**Trait**^**2**^				
		**DSB**	**DGL**	**MCE**	**MSB**	**MGL**
DCE	0.03 (0.01)*	0.37 (0.17)*	0.50 (0.08)*	−0.27 (0.22)	−0.16 (0.22)	−0.22 (0.16)
DSB	0.02 (0.00)*		−0.08 (0.12)	−0.22 (0.31)	−0.88 (0.20)*	−0.24 (0.24)
DGL	0.41 (0.02)*			0.04 (0.14)	−0.30 (0.19)	0.01 (0.08)
MCE	0.02 (0.01)				0.67 (0.19)*	0.13 (0.18)
MSB	0.02 (0.01)*					−0.06 (0.25)
MGL	0.07 (0.01)*					

^*^P < 0.05; ^1^heritabilities and genetic correlations ^2^DCE = direct calving ease, DSB = direct stillbirth, DGL = direct gestation length, MCE = maternal calving ease, MSB = maternal stillbirth, MGL = maternal gestation length; standard errors are indicated in brackets.

**Table 4:** **Genetic parameters**^**1 **^**for calving ease, stillbirth and gestation length between parities and within traits**

**Trait**^**2**^			**Direct**		**Maternal**	
			**First**	**Later**	**First**	**Later**
CE	Direct	First	0.11 (0.022)			
		Later	0.80 (0.119)	0.03 (0.006)		
	Maternal	First	−0.47 (0.130)	−0.12 (0.174)	0.08 (0.019)	
		Later	−0.40 (0.215)	−0.28 (0.195)	0.84 (0.150)	0.02 (0.007)
SB	Direct	First	0.016 (0.01)			
		Later	-	0.017 (0.01)		
	Maternal	First	0.57 (0.47)	-	0.024 (0.01)	
		Later	-	−0.88 (0.20)	-	0.011 (0.01)
GL	Direct	First	0.30 (0.024)			
		Later	0.96 (0.022)	0.38 (0.017)		
	Maternal	First	0.01 (0.119)	0.13 (0.109)	0.05 (0.013)	
		Later	−0.22 (0.101)	−0.04 (0.088)	0.82 (0.125)	0.05 (0.011)

^1^heritabilities (diagonals) and genetic correlations (off-diagonals); ^2^CE = calving ease, SB = stillbirth, GL = gestation length; standard errors are indicated in brackets.

**Table 5:** Variances and genetic parameters for calving ease, stillbirth and gestation length from first parity univariate models

**Trait**^**1**^**Model**^**2**^	**Variance components and genetic parameters**^**3**^							
	***σ***_***P***_^**2**^	***σ***_***Ad***_^**2**^	***σ***_***Am***_^**2**^	***σ***_***TBV***_^**2**^	***σ***_***e***_^**2**^	***h***_***d***_^**2**^	***h***_***m***_^**2**^	***r***_***dm***_
CE^1^								
S-MGS^2^	0.464 (0.01)	0.050 (0.01)	0.024 (0.01)	0.048 (0.01)	0.443 (0.01)	0.108 (0.02)	0.051 (0.02)	−0.373 (0.15)
A1^2^	0.464 (0.01)	0.055 (0.01)	0.022 (0.01)	0.041 (0.01)	0.404 (0.01)	0.119 (0.02)	0.048 (0.01)	−0.523 (0.13)
A2^2^	0.462 (0.01)	0.054 (0.01)	0.022 (0.01)	0.046 (0.01)	0.401 (0.01)	0.117 (0.02)	0.033 (0.01)	−0.444 (0.13)
SB^1^								
S-MGS^2^	0.096 (0.001)	0.002 (0.001)	0.002 (0.001)	0.006 (0.001)	0.094 (0.001)	0.016 (0.01)	0.024 (0.01)	0.567 (0.47)
A1^2^	0.097 (0.001)	0.002 (0.001)	0.002 (0.001)	0.006 (0.002)	0.092 (0.001)	0.017 (0.01)	0.018 (0.01)	0.704 (0.75)
A2^2^	0.095 (0.001)	0.002 (0.001)	0.002 (0.001)	0.006 (0.002)	0.090 (0.001)	0.019 (0.01)	0.022 (0.01)	0.623 (0.62)
**GL**^**1**^								
S-MGS^2^	24.30 (0.51)	12.06 (1.31)	1.99 (0.50)	12.35 (1.25)	18.95 (0.20)	0.496 (0.05)	0.081 (0.02)	−0.172 (0.12)
A1^2^	23.50 (0.42)	13.22 (1.41)	1.65 (0.45)	12.85 (1.19)	9.64 (0.85)	0.563 (0.05)	0.070 (0.02)	−0.216 (0.11)
A2^2^	23.32 (0.37)	11.66 (1.20)	1.67 (0.41)	11.86 (0.96)	10.73 (0.71)	0.499 (0.05)	0.072 (0.02)	−0.166 (0.11)

^1^CE = calving ease, SB = stillbirth, GL = gestation length; ^2^ S-MGS = sire-maternal grandsire model, A1 = animal model 1 excludes animals recorded at both birth and calving, A2 = animal model includes all records; ^3^*σ*_*P*_^2^ = phenotypic, *σ*_*Ad*_^2^ = additive genetic direct; *σ*_*Am*_^2^ = additive genetic maternal; *σ*_*TBV*_^2^= total breeding value; *σ*_*e*_^2^ = environmental; *h*_*d*_^2^ = direct; *h*_*m*_^2^= maternal heritability; *r*_*dm*_ = genetic direct-maternal correlation; standard errors are indicated in brackets.

**Table 6:** **Accuracies of prediction (r) of average first parity PTA from 25 young**^**1 **^**and older**^**2**^**sires**

	**Young sires**^**1**^		**Older sires**^**2**^	
	**Direct**	**Maternal**	**Direct**	**Maternal**
Average progeny group size	88	12	122	101
Model^3^	r	R	r	r
**CE**^**4**^				
Univariate S-MGS^5^	0.67	0.46	0.68	0.61
Univariate A1	0.71	0.50	0.72	0.60
Bivariate with GL	0.70	0.52	0.71	0.60
Bivariate with SB	0.71	0.49	0.72	0.64
Bivariate between parities	0.77	0.50	0.82	0.66
**GL**^**4**^				
Univariate S-MGS^5^	0.77	0.69	0.83	0.75
Univariate A1	0.85	0.45	0.86	0.59
Bivariate with CE	0.86	0.46	0.87	0.59
Bivariate with SB	0.84	0.46	0.86	0.56
Bivariate between parities	0.90	0.78	0.93	0.87
**SB**^**4**^				
Univariate S-MGS^5^	0.31	0.34	0.51	0.54
Univariate A1	0.39	0.40	0.49	0.48
Bivariate with CE	0.55	0.42	0.65	0.54
Bivariate with GL	0.38	0.46	0.51	0.57
Bivariate between parities	-	-	-	-

^1^born between 1999 and 2002; ^2^born between 1990 and 1994; ^3^ S-MGS = sire-maternal grandsire model, A1 = animal model 1, excludes animals recorded at both birth and calving; ^4^CE = calving ease, GL = gestation length, SB = stillbirth; ^5^for S-MGS models, values are based on sire and maternal grandsire variances.

### Genetic parameters

#### Heritabilities

Table 2 and 3 show the estimates of heritabilities and genetic correlations among traits obtained from bivariate animal models in first and later parities and Table 4 presents parameter estimates obtained by between-parity models for each trait respectively. Therefore, results for later parities in Table 4 account for selection based on first parity, whereas results in Table 3 do not. All heritabilities estimated for CE, SB and GL were within the range of previously published estimates of these traits in dairy cattle [16,23,33]. Heritabilities of CE were low (direct: 12% first parity and 3% later parities; maternal: 5% first parity and 2% later parities) and the direct heritability was approximately twice as large as the maternal heritability. GL appeared moderately heritable, with the direct heritability (57% first parity and 41% later parities) being considerably larger than the maternal heritability (7% first parity and 7% later parities). This supports the view that the genetic variation of this trait lies primarily in the triggering of parturition by the foetus [22,33,34] rather than in the maternal response to this trigger. All heritabilities were larger in first parity than in later parities, as reported elsewhere in the literature [7,23]. This supports the general assumption that the variation in calving performance is larger in heifers than in cows [24,35]. In addition, heritability estimates are frequency dependent when applying linear models to categorical traits.

Both direct and maternal heritabilities for SB were low, irrespectively of parity, with the direct heritability ranging from 1.8% to 2.0% (not significant in first parity) and the maternal heritability ranging from 2.0% to 3.2%. These estimates agree with values from the literature, which range from 1.6% to 10% for direct heritability and from 2.0% to 13% for maternal heritability [22,32-34,36,37].

### Direct and maternal genetic correlations

#### Within traits and within parities

The estimated genetic direct-maternal correlations for CE and GL presented in Table 2,3 and 4 were low to moderate (−0.52 to −0.22). For GL, the direct-maternal correlations were not significantly different from zero. For CE, a significant genetic relationship between the direct and maternal effects in first parity (−0.53) was detected. This negative direct-maternal correlation suggests that animals with a lower genetic risk of being born with difficulties are genetically prone to have more difficulty at first calving. Numerous studies confirm a negative genetic relationship between the direct and maternal effect of CE [38-40], although positive correlations also appear in the literature [23]. The negative genetic correlation between direct and maternal effects of CE implies that dairy farmers need to base selection decisions on both the direct and the maternal PTA of a sire for CE in first parity, to avoid long-term negative consequences. An optimum index value for genetic merit in CE is therefore preferable, as discussed later.

Due to very low heritabilities and very high standard errors, the estimated direct-maternal genetic correlation of SB, obtained by the different models, were not informative. Studies estimating this covariance in large datasets (> 400 000) report correlations close to zero [20,23,38], although with considerable standard errors. To date, there is no clear evidence to recommend a change from the common practice of assuming this covariance as equal to zero.

#### Between traits

Table 2 shows the estimated genetic correlations between the direct and maternal effects of CE, SB and GL in first parity heifers obtained from bivariate analyses. Table 3 shows the estimates of the same models for later parity cows (parity 2 and 3). In general, CE and SB were strongly genetically correlated, whereas the relationships of GL with CE and SB were weak to moderate. Both the direct and maternal correlations between CE and SB were positive and high in first parity (0.84; 0.85), and positive and moderate in later parities (0.37; 0.67). This suggests that both difficult birth and difficult calving are genetically associated with a higher frequency of direct and maternal stillbirth respectively, regardless of parity. The findings for the UK dataset thereby support the consensus of a strong genetic relationship between CE and SB [20,37-41]. In this study, correlations of CE and SB were not significantly different from 1. However, Hansen et al. [20] and Cervantes et al. [41] provide evidence of genetic distinctiveness for these traits, with estimates of similar magnitude to those from this study, but with smaller standard errors. Furthermore, a simple meta-analysis [31] pooling estimates from this study and four other studies [20,21,41,42] results in a direct and maternal genetic correlations of between CE and SB of 0.79 ± 0.02 and 0.65 ± 0.03, respectively, which suggests a genetic distinctiveness of these traits.

The genetic correlations between GL and the calving traits differed between parities (Table 2 and 3). A moderate positive genetic correlation (0.65) was found between maternal GL and maternal SB in first parity. This suggests that an individual with a longer than average gestation period is genetically more likely to give birth to a stillborn calf in first parity and *vice versa*. Genetic correlations between GL and SB in later parities were not significant (Table 3). A direct genetic relationship between GL and CE was detected, but only in later parities. No maternal relationship was detected in later parities. The direct effect of GL was found to be moderately correlated to the direct effect of CE. This positive correlation (0.50) between direct CE and direct GL suggests that a calf that gestates longer before birth to a multiparous dam is genetically prone to a difficult birth and *vice versa*. Similar positive correlations between direct GL and direct CE are reported in beef cattle [41], Danish Holstein cattle [20] and UK Holstein cattle [43], and support the findings from the UK dataset here. However, in this study, the genetic correlation between maternal GL and maternal CE was non-significant, although this relationship is generally reported to be low to moderate [20,21].

All relationships between direct effects of one trait and maternal effects of the other trait (and *vice versa*) were non-significant, except for the genetic correlation between direct SB and maternal CE in first parity, which was high and positive. This specific relationship is difficult to estimate at the animal level and the high estimate may be due to the inaccuracy of the SB variance components. In general, studies in the literature report non-significant genetic correlations between the genetic direct effects and the genetic maternal effects between traits [20,25,41,44].

#### Between parities within traits

Table 4 presents the genetic parameters estimated by the bivariate S-MGS models that treat first and later parity records as correlated traits. Estimated genetic correlations between first and later parities were 0.80 ± 0.12 for direct CE and 0.84 ± 0.15 for maternal CE. These estimates are similar to those estimates obtained by the threshold model reported by Wiggans et al., [24] but slightly higher than those reported in general [23,37,41]. Among the studies estimating genetic correlations of CE between parities, there is general agreement that both direct and maternal CE are genetically distinct traits in first and later parities, which suggests that both ease of birth and ease of calving represent a different trait in heifers and in cows [23,24,41]. However, the standard errors reported here are too large to infer genetic distinction between first and later parities from this study alone.

Direct and maternal GL are rarely considered separately in studies that estimate between-parity correlations. Table 4 shows that different between-parity genetic correlations are found for direct GL and maternal GL. This emphasizes the fact that direct and maternal GL are separate traits, and thus must be analysed and interpreted with this in mind. For direct GL, the estimated correlation between first and later parities was near unity (0.96 ± 0.02) but the same correlation for maternal GL was lower (0.82 ± 0.13). However, in this case too, the standard error is too large to conclude that maternal GL is a distinct trait in first and later parities. Other studies on larger datasets show a correlation that is high but nevertheless significantly different from 1 [45,46] which implies that maternally, the gestation length of a heifer and a cow are genetically distinct traits.

When fitting a between-parity S-MGS model for SB, results were difficult to obtain. Other analyses, using univariate and bivariate models, already showed the difficulty of obtaining an accurate estimate of the direct-maternal genetic correlation for SB within parities. With the between-parity model, the likelihood surface was practically flat which hampered convergence to sensible estimates.

#### Total heritable variance

The total heritable variance gives a holistic measure of the genetic variance affecting calving and accounts for both the maternal and direct sources of variance. Although some estimates in this study were inaccurate (in particular the direct-maternal genetic covariance for SB), the estimates of *σ*_*TBV*_^2^ presented in Table 5 show how the maternal variance and direct-maternal genetic covariance contribute to the total genetic variance. Focusing on animal model A2, the total variance was smaller than the direct variance for CE and GL by 26% and 3%, respectively, although these differences were not significant. For SB, the very large and positive direct-maternal genetic covariance, in combination with the small direct variance, caused *σ*_*TBV*_^2^ to be by ~ 400% larger than the additive direct variance.

When a farmer makes a selection decision based on a maternally affected trait, population mean performances change in response to both its direct and maternal breeding value. The TBV_*i*_ as described by Bijma et al. [26] is suggested to represent the total additive value of an individual. However, it does not represent the impact of that individual on the population mean since this impact will depend on the time period and the frequency of expression of the direct and maternal effects in the population within that period. Gene flow methodology [47,48] shows that contributions of the direct and maternal effects to genetic change in calving traits depend on several factors which determine how often the maternal effect is expressed, e.g. how many calvings, how many calves are kept as replacement heifers and the breeding system (pure breeding or crossbreeding). Therefore, while theoretically TBV_*i*_ and *σ*_*TBV*_^2^ show the importance of considering maternal effects and their interrelationship with direct effects, practically, an index value that is not the simple sum and represents the total impact of an individual would be useful to farmers. This would be in addition to the separate direct and maternal EBV that are already provided.

### Model comparison

In this section, animal models are compared to S-MGS models, and univariate models to bivariate models. Furthermore, the benefit in treating first and later-parity calvings as correlated traits in a bivariate between-parity model is discussed. A potential bias due to an environmental direct-maternal covariance is also evaluated. All models that are discussed are linear models. Several studies have explored the advantages of threshold models over linear models for the analysis of calving traits [49,50] given that according to the categorical nature of the traits, threshold models should theoretically be superior, as explained by Gianola [51]. Findings show that computational requirements are greater for threshold compared to linear models and Monte Carlo methods needed to obtain the most reliable parameter estimates. However, software that can estimate variances without relying on Monte Carlo simulation methods, e.g. through the use of approximations to maximum likelihood in complex GLMM is available but limited [52]. For calving traits, comparisons between linear and threshold models have shown very high correlations between PTA, meaning that the ranking of sires is not greatly influenced by the use of a linear model [35,49,53]. Threshold models have been shown to take specific interactions into account which can potentially be problematic for linear models [54,55].

#### Animal model versus S-MGS model

Table 5 contains the results of the univariate analyses on first parity data and compares animal model A1 with the S-MGS model, since neither of these two models are expected to show bias due to the ignored environmental direct-maternal covariances discussed in the Methods, unlike animal model A2.

Table 5 shows that, between traits, direct heritabilities and phenotypic variances when estimated by A1 and S-MGS models were very similar but the residual variances were consistently larger for S-MGS models compared to both A1 and A2 models. The residual variance of an S-MGS model contains the default environmental variance plus a Mendelian sampling term and the remaining unexplained additive variance terms from dams totalling 916σAd2+34σAm2+34σAdm. Accuracies of PTA for young and older sires are presented in Table 6. Comparison of the S-MGS model with A1 and A2 models shows that there was only a small loss in accuracy when fitting the S-MGS model. In some cases, mainly for older sires for which more progeny information is available, there was an increase in accuracy of PTA when the S-MGS model was fitted as opposed to the animal model. This is probably due to the slightly higher heritabilities that were estimated by the S-MGS model (see Table 5). The computation time required with univariate animal models was 10 times greater than with univariate S-MGS models. Furthermore, when increasing the model complexity, animal models failed to converge, whereas S-MGS models were robust. The between-parity model in this study was an example where animal models failed, whereas S-MGS models performed well. Advantages in computation time and versatility of the S-MGS model therefore compensate well for the slight loss in accuracy of any resulting estimates.

#### Potential bias in the direct-maternal genetic correlation

Table 5 shows the estimated genetic direct-maternal correlations within traits for first parity data, when applying two univariate animal models. As described earlier, part of the data corresponding to specific dam-offspring pairs was deleted from the dataset (7.8%) to remove for a potential environmental direct-maternal covariance. In Table 5, animal model A1 represents the analysis of the edited data, whereas A2 represents the analysis of the complete data. Comparison of the results for A1 and A2 models shows that deleting records on dam-offspring pairs had only a small and non-significant impact on direct-maternal genetic correlations. The observed change implies that the environmental direct-maternal covariance was negative and small in this dataset. Since estimates of the direct-maternal genetic correlation do not differ significantly, the magnitude of the environmental covariance in this dataset is likely to be negligible and changes observed could be due to chance alone.

#### Univariate versus bivariate models

**Figure 1:**
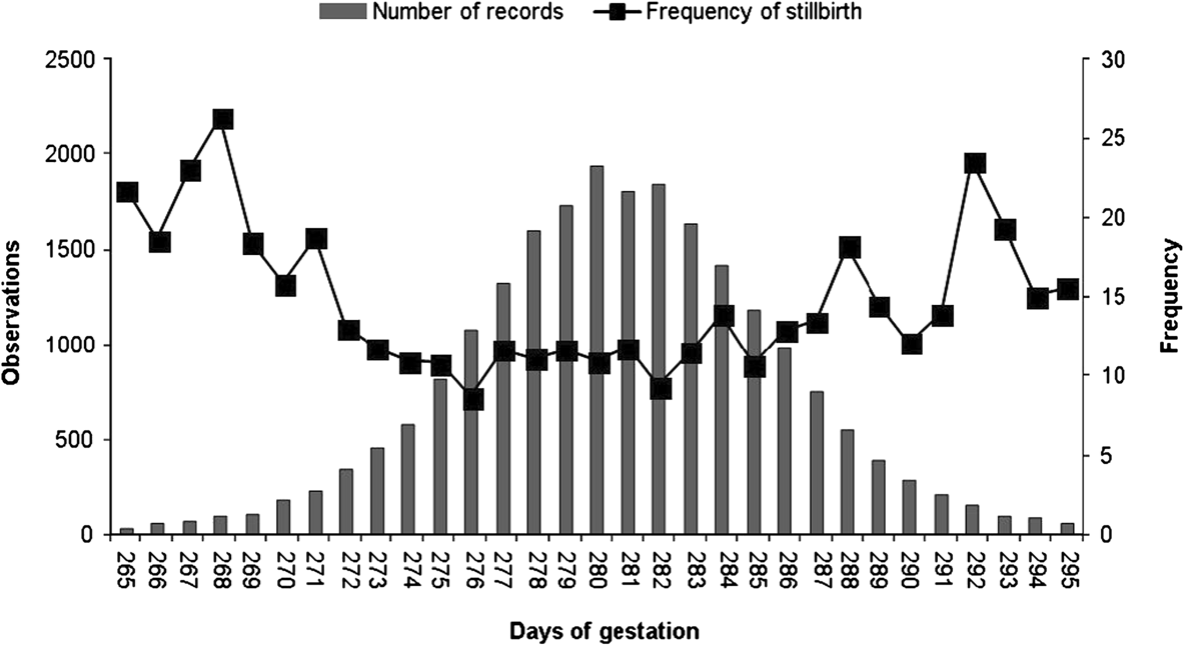
Phenotypic relationship between gestation length and stillbirth.

**Figure 2:**
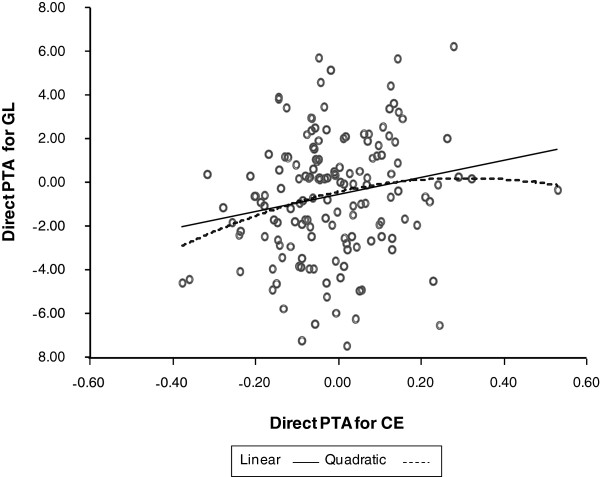
Association of direct PTA obtained from univariate models between gestation length and calving ease.

**Figure 3:**
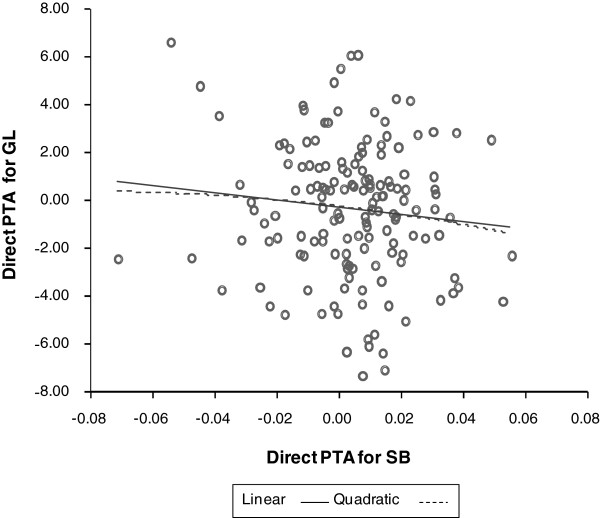
Association of direct PTA obtained from univariate models between gestation length and stillbirth.

One of the important points when using GL as an indicator trait for the analyses of calving traits is its potential non-linear relationship with CE and SB. The relationship of GL with both SB and CE was clearly non-linear on a phenotypic scale (Figure 1, first parity). However, a visual assessment of plotted EBV obtained from univariate first parity animal models showed that relationships were not better approximated by a quadratic relationship (quadratic regression coefficients P > 0.05) than by a linear relationship. Figure 2 and 3 show this for 150 sires with > 25 progeny. Thus, it was concluded that quadratic relationships between GL, CE and SB were not detected and that, for this study and under the assumption that relationships of higher polynomial degree would be unlikely, the use of GL as indicator trait in linear bivariate models was justified.

Table 6 demonstrates that, for calving traits, bivariate models are slightly superior to univariate traits. In particular, the maternal variance of low heritable traits (CE and SB) benefitted from including a correlated trait in the model. Accuracies of direct PTA also showed a slight improvement from bivariate analysis, in particular for the low heritable SB trait. Estimates obtained for SB heritabilities with the univariate and bivariate models (Table 5 and 2) demonstrate that these models provided nearly identical estimated direct variances, although the maternal variance showed a small but significant higher estimate with bivariate analyses. Strong genetic correlations were found between CE and SB, SB and GL, and CE and GL, which are likely to explain the increase in accuracy of PTA obtained by the bivariate model compared to the univariate model. The maternal variance of the indicator trait, GL, also benefitted from the bivariate model compared to the univariate model, although the accuracy of the maternal PTA of GL was slightly decreased with the bivariate model. Genetic direct-maternal correlations for CE and GL showed little change between univariate and bivariate models, while the estimate of this correlation for SB showed considerably more change but is too imprecise to be interpreted.

#### Inclusion of later parities

Calving traits in first and later parities were highly correlated, which results in a considerably greater predictive ability of PTA for all traits when later parity information is added as a correlated trait to the model (Table 6). Accuracies increased, for both direct and maternal PTA of CE and GL, when compared to the univariate model.

## Conclusions

Heritabilities for CE, SB and GL in UK Holstein cattle were in the range of previously reported genetic parameters for these traits. Both the direct and maternal genetic variances were considerably lower in cows than in heifers. Direct and maternal effects of CE were negatively correlated but this was established only in first parity. CE and SB were genetically highly correlated traits for both direct and maternal components, especially in first parity. GL showed a moderate relationship with CE and SB, which differed between parities but implies that genetically longer gestations are associated with reduced calving performance. The three traits all had high and positive genetic correlations between parities but parities were not demonstrated as being genetically distinct for any trait with the data available. Different between-parity genetic correlations estimated for direct GL and maternal GL emphasize that these are separate traits and thus should be treated as such. Estimates of *σ*_*TBV*_^2^ indicate that the total additive genetic variance in a calving trait may be lower than the additive direct variance when the genetic direct-maternal covariance is highly negative and the additive maternal variance is small.

Results from this study further demonstrated that estimating genetic parameters for calving traits is complex. Developing a statistical model for a maternally affected trait requires a careful balance between sufficient predictive ability and computational practicality, which in turn are affected by the size of the dataset, potential biases in data recording, the trait in question, computational facilities and the amount of time in hand. However, in general, PTA estimates for calving traits benefitted from multi-trait models. Furthermore, estimates were only slightly less accurate when a S-MGS model was fitted instead of an animal model. With the current computing facilities, S-MGS models exceeded animal models in terms of practicality, as their robustness allowed the analysis of more data and the inclusion of more traits e.g. information from later parities. In the genetic evaluation of calving traits genetic correlations between traits and between parities need to be estimated and the direct-maternal genetic correlation must be considered with caution.

## Competing interests

The authors declare that they have no competing interests.

## Authors’ contributions

SAE, JAW, EW and MPC conceived and designed the study. SAE, JAW and EW designed the models and SAE analysed the data. SAE, JAW, EW and MPC interpreted the results, and SAE, JAW, EW and MPC wrote the paper. All authors read and approved the final manuscript.

## Supplementary Material

Additional file 1 **Herd year variances, permanent environmental variances and prediction error variances.** The estimated herd-year and permanent environmental variances from first and later parity univariate and bivariate models plus the prediction error variances of average first parity PTA from 25 young sires born between 1999 and 2006 and 25 older sires born between1990 and 1998. Click here for file
