# Supplementary material for: Evaluating alternate models to estimate genetic parameters of calving traits in United Kingdom Holstein-Friesian dairy cattle
Source: Genet Sel Evol. 2012 Jul 28;44(1):23. doi: 10.1186/1297-9686-44-23 (PMC3468354; doi:10.1186/1297-9686-44-23)
Supplement: Additional file 1 — Herd year variances, permanent environmental variances and prediction error variances. The estimated herd-year and permanent environmental variances from first and later parity univariate and bivariate models plus the prediction error variances of average first parity PTA from 25 young sires born between 1999 and 2006 and 25 older sires born between1990 and 1998. [file 1297-9686-44-23-S1.pdf]

Table S1 - Herd-year variances for calving ease, stillbirth and gestation length from first parity univariate and bivariate models between traits

			Parameter
	Trait[1] and model[2]		σ_{hy}^2
Univariate first parity	CE		
		S-MGS model[2]	0.037 (0.003)
		A1[2]	0.038 (0.003)
		A2[2]	0.037 (0.003)
	SB		
		S-MGS model[2]	0.002 (0.0001)
		A1[2]	0.003 (0.0001)
		A2[2]	0.002 (0.0001)
	GL		
		S-MGS model[2]	0.706 (0.13)
		A1[2]	0.677 (0.14)
		A2[2]	0.685 (0.13)
Bivariate first parity	CE		
		SB	0.034 (0.003)
		GL	0.034 (0.003)
	SB		
		CE	0.002 (0.001)
		GL	0.003 (0.001)
	GL		
		CE	0.673 (0.14)
		SB	0.686 (0.14)

[1]CE = calving ease, GL = gestation length, SB = stillbirth; [2]S-MGS= sire-maternal grandsire model, A1 = animal model 1 excludes animals recorded at birth and calving, A2 = animal model includes all records;
[3] σ_{hy}^2 = herd-year; standard errors are indicated in brackets

Table S2 – Herd-year and permanent environmental variances for calving ease, gestation length and stillbirth from later parity bivariate animal models between traits[1]

	Trait[2] and model		Parameter σ^2_{hy}	Parameter σ^2_{pe}
Bivariate later parity	CE			
		SB	0.019 (0.001)	0.017 (0.006)
		GL	0.019 (0.001)	0.017 (0.006)
	SB			
		CE	0.005 (0.0003)	0.0003 (0.0006)
		GL	0.005 (0.0003)	0.0003 (0.0006)
	GL			
		CE	0.508 (0.06)	1.167 (0.33)
		SB	0.508 (0.06)	1.167 (0.33)

[1] A1 = animal model 1 excludes animals recorded at birth and calving; [2]CE=calving ease, GL=gestation length, SB=stillbirth; standard errors are indicated in brackets

Table S3 - Prediction error variances of average first parity PTA's from 25 young[1] and older[2] sires

	Young sires[1]		Older sires[1]	
	Direct	Maternal	Direct	Maternal
Average progeny group size	88	12	122	101
Model[3]	PEV	PEV	PEV	PEV
CE[4]				
Univariate S-MGS[5]	0.0066	0.0045	0.0066	0.0035
Univariate A1	0.0261	0.0151	0.0256	0.0135
Bivariate with GL	0.0263	0.0162	0.0266	0.0141
Bivariate with SB	0.0263	0.0132	0.0257	0.0102
Bivariate across parities	0.0689	0.0051	0.0042	0.0038
GL[4]				
Univariate S-MGS[5]	1.1766	1.1260	0.9087	0.4002
Univariate A 1	3.3837	0.5399	3.2163	1.0350
Bivariate with CE	3.2819	1.2506	3.1916	1.0239
Bivariate with SB	3.6005	1.1877	3.1297	1.0239
Bivariate across parities	0.2367	0.1987	0.1615	0.1287
SB[4]				
Univariate S-MGS[5]	0.0004	0.0008	0.0003	0.0007
Univariate A1	0.0014	0.0015	0.0012	0.0012
Bivariate with CE	0.0004	0.0018	0.0365	0.0015
Bivariate with GL	0.0008	0.0025	0.0255	0.0021
Bivariate across parities	-	-	-	-

[1] born between 1999 and 2002; [2] born between 1990 and 1994; [3]S-MGS=sire-maternal grandsire model, A1 = animal model 1, excludes animals recorded at both birth and calving; [4]CE = calving ease, GL = gestation length, SB = stillbirth; [5]For S-MGS models, values are based on sire and maternal grandsire variances
